# Decoding physiological and pathological roles of innate immune cells in eye diseases: the perspectives from single-cell RNA sequencing

**DOI:** 10.3389/fimmu.2024.1490719

**Published:** 2024-10-31

**Authors:** Chen Lu, Xiying Mao, Songtao Yuan

**Affiliations:** Department of Ophthalmology, The First Affiliated Hospital of Nanjing Medical University, Nanjing, China

**Keywords:** single-cell RNA sequencing, innate immunity, retina, microglia, monocyte, neutrophil, eye diseases

## Abstract

Single-cell RNA sequencing (scRNA-seq) has facilitated a deeper comprehension of the molecular mechanisms behind eye diseases and has prompted the selection of precise therapeutic targets by examining the cellular and molecular intricacies at the single-cell level. This review delineates the pivotal role of scRNA-seq in elucidating the functions of innate immune cells within the context of ocular pathologies. Recent advancements in scRNA-seq have revealed that innate immune cells, both from the periphery and resident in the retina, are actively engaged in various stages of multiple eye diseases. Notably, resident microglia and infiltrating neutrophils exhibit swift responses during the initial phase of injury, while peripheral monocyte-derived macrophages exhibit transcriptomic profiles akin to those of activated microglia, suggesting their potential for long-term residence within the retina. The scRNA-seq analyses have underscored the cellular heterogeneity and gene expression alterations within innate immune cells, which, while sharing commonalities, exhibit disease-specific variations. These insights have not only broadened our understanding of the cellular and molecular mechanisms in eye diseases but also paved the way for the identification of candidate targets for targeted therapeutic interventions. The application of scRNA-seq technology has heralded a new era in the study of ocular pathologies, enabling a more detailed appreciation of the roles that innate immune cells play across a spectrum of eye diseases.

## 1 Introduction

The innate immune system serves as the first line of defense against pathogens and injuries. Innate immune cells such as microglia, monocyte-derived macrophages, and neutrophils are pivotal for the maintenance of retinal homeostasis and the formation of several inflammatory retinal disorders (1). The activation of innate immune cells is influenced by the ocular microenvironment, and traditional histological studies and inflammatory cytokine/chemokine examinations have confirmed their roles in eye diseases (2–6). Those innate immune cell-associated cytokines/chemokines can predict disease activity, some of which have become precise targets for treatment (7, 8). However, the function of each innate immune cell and their interactions remain complex and unclear. Dissecting their individual roles in pathogenesis is of great significance, as waves of innate immune response in the retina remain intricate under disease context (9–11).

The development of single-cell RNA sequencing (scRNA-seq) has already contributed to profound discoveries. Compared with traditional bulk RNA sequencing (RNA-seq), scRNA-seq maps transcriptome to an individual cell, which has an edge on unveiling cellular composition differences among samples and assaying altered gene expression at the single-cell level, especially in studying complex tissues composed of many distinct cell types that are typically difficult to dissect experimentally like brain tissues and the retina (12, 13). The landmark study of scRNA-seq applied in ophthalmology began with murine retina profiled with Drop-seq barcoding strategy (14). Thereafter, a series of studies using scRNA-seq created a comprehensive transcriptome atlas of eye structures, including the cornea, aqueous humor, ciliary body, choroid, and retina (15–21). These available online public scRNA-seq data make it convenient for researchers to compare datasets to validate new outcomes (**Table 1**). In this review, we systematically summarized the roles of innate immune cells in cellular and molecular mechanisms behind several common eye diseases from the perspectives of scRNA-seq.

**Table 1:** Studies for eye diseases applying scRNA-seq.

Title	Year	Species	Tissue	Associated-eye disease	Accession number	Reference
Single-cell transcriptomics of the human retinal pigment epithelium and choroid in health and macular degeneration	2019	Human	Choroid, RPE	AMD	GSE135922	(21)
Single-cell transcriptomic atlas of the human retina identifies cell types associated with age-related macular degeneration	2019	Human	Retina	AMD	GSE137537	(66)
Single-cell analysis reveals inflammatory interactions driving macular degeneration	2023	Human	Retina	AMD	GSE221042	(38)
Mapping the origin and fate of myeloid cells in distinct compartments of the eye by single-cell profiling	2021	Mouse	Retina, cornea, and ciliary body	AMD	GSE160797	(34)
Implication of specific retinal cell-type involvement and gene expression changes in AMD progression using integrative analysis of single-cell and bulk RNA-seq profiling	2021	Human	Retina	AMD	GSE155288	(67)
As in Real Estate, Location Matters Cellular Expression of Complement Varies Between Macular and Peripheral Regions of the Retina and Supporting Tissues	2021	Human	Retina	AMD	GSE188280	(28)
Single-cell transcriptome analysis of the Akimba mouse retina reveals cell-type-specific insights into the pathobiology of diabetic retinopathy	2020	Mouse	Retina	DR	E-MTAB-9061	(32)
Single cell RNA sequencing (scRNA-Seq) deciphering pathological alterations in streptozotocin-induced diabetic retinas	2021	Mouse	Retina	DR	GSE178121	(33)
Single-Cell Transcriptome Profiling Reveals the Suppressive Role of Retinal Neurons in Microglia Activation Under Diabetes Mellitus	2021	Macaque	Retina	DR	GSE168908	(35)
Pathogenesis Study Based on High-Throughput Single-Cell Sequencing Analysis Reveals Novel Transcriptional Landscape and Heterogeneity of Retinal Cells in Type 2 Diabetic Mice	2021	Mouse	Retina	DR	PRJNA653629	(29)
Single-Cell Transcriptomics Reveals Novel Role of Microglia in Fibrovascular Membrane of Proliferative Diabetic Retinopathy	2022	Human	Fibrovascular membrane	DR	GSE165784	(42)
Single-cell transcriptomics analysis of proliferative diabetic retinopathy fibrovascular membranes reveals AEBP1 as fibrogenesis modulator	2023	Human	Fibrovascular membrane	DR	GSE245561	(45)
Single−cell RNA sequencing reveals roles of unique retinal microglia types in early diabetic retinopathy	2024	Rat	Retina–RPE–choroid complex	DR	GSE209872	(36)
Neutrophil extracellular traps target senescent vasculature for tissue remodeling in retinopathy	2020	Mouse	Retina	OIR	GSE150703	(56)
A specific RIP3+ subpopulation of microglia promotes retinopathy through a hypoxia-triggered necroptotic mechanism	2021	Mouse	Retina	OIR	GSE152928	(68)
eNOS controls angiogenic sprouting and retinal neovascularization through the regulation of endothelial cell polarity	2021	Mouse	Retina	OIR	GSE174400	(69)
Pathological angiogenesis in retinopathy engages cellular senescence and is amenable to therapeutic elimination via BCL-xL inhibition	2021	Mouse	Retina	OIR	GSE158799	(70)
Single-cell transcriptome analyses reveal microglia types associated with proliferative retinopathy	2022	Mouse	Retina	OIR	GSE199792	(31)
Cell atlas of aqueous humor outflow pathways in eyes of humans and four model species provides insight into glaucoma pathogenesis	2020	Human, macaque, mouse, pig	Trabecular meshwork	Glaucoma	GSE146188	(17)
Molecular taxonomy of human ocular outflow tissues defined by single-cell transcriptomics	2020	Human	Trabecular meshwork	Glaucoma	PRJNA616025	(18)
Single-cell transcriptome analysis of regenerating RGCs reveals potent glaucoma neural repair genes	2022	Mouse	Retinal ganglion cells	Glaucoma	GSE206625	(71)
Single-Cell Profiles of Retinal Ganglion Cells Differing in Resilience to Injury Reveal Neuroprotective Genes	2019	Mouse	Retina	ONC	GSE137400	(72)
Immune stimulation recruits a subset of pro−regenerative macrophages to the retina that promotes axonal regrowth of injured neurons	2023	Mouse	Retina	ONC	GSE232470	(49)
Temporal single-cell atlas of non-neuronal retinal cells reveals dynamic, coordinated multicellular responses to central nervous system injury	2023	Mouse	Retina, RPE	ONC	GSE199317	(11)
Comprehensive analysis of a mouse model of spontaneous uveoretinitis using single-cell RNA sequencing	2019	Mouse	Retina	Uveoretinitis	GSE132229	(73)
Intraocular dendritic cells characterize HLA-B27-associated acute anterior uveitis	2021	Human	Aqueous humor	Uveitis	GSE178833	(60)
Genetic landscape and autoimmunity of monocytes in developing Vogt-Koyanagi-Harada disease	2020	Human	Peripheral blood	VKH disease	GSE148020	(50)
Sex-specific circulating unconventional neutrophils determine immunological outcome of autoinflammatory Behçet’s uveitis	2024	Human	Peripheral blood	Behçet’s uveitis	HRA002148	(55)
IL-11 Is Elevated and Drives the Profibrotic Phenotype Transition of Orbital Fibroblasts in Thyroid-Associated Ophthalmopathy	2022	Human	Orbital adipose/connective tissues	TAO	GSE194324	(74)

AMD, age-related macular degeneration; DR, diabetic retinopathy; OIR, oxygen-induced retinopathy; ONC, optic nerve crush; VKH disease, Vogt–Koyanagi–Harada disease; TAO, thyroid-associated ophthalmopathy.

## 2 Microglia

Residing in the inner part of the neuronal retina, microglia, a specialized type of yolk sac-derived macrophage, can switch from a resting state to an activated state and work in surveillance and maintenance of homeostasis in the retina (22, 23).

Multiple types of proliferative retinopathy, such as proliferative diabetic retinopathy (PDR) and wet age-related macular degeneration (AMD), are relevant to the hyperactivation of microglia (24–27). ScRNA-seq performed on the retina and choroid from postmortem eyes found that microglia play an important role in early AMD (28). There are an increasing number of studies performing scRNA-seq on rodent models with retinal neovascularization, validating functions of microglia in proliferative retinopathy (29–34).

### 2.1 Microglia predominating in pathogenic cell interactions in DR

ScRNA-seq was performed on the retina of cynomolgus monkeys with non-proliferative diabetic retinopathy (NPDR) to assess the suppressive role of retinal microglia in diabetic retinopathy (DR) (35). Among the cell atlas, microglia exhibited the largest number of upregulated genes under diabetes, showing that hyperglycemia induced microglial activation and elevated inflammation levels. CellChat analysis found that microglia were the major source of TNF-α. Likewise, in a streptozotocin (STZ)-induced diabetic rat model, scRNA-seq data analysis found an inflammatory regulatory network centered on microglia in early DR (36). Here, M1 microglia were distinguished from M2 microglia by the different expression levels of CCR5, and M1 microglia were further divided into EGR2^+^ M1 microglia and EGR2^−^ M1 microglia, the former of which showed a greater proinflammatory effect and participated in the regulation of retinal vascular permeability in the early stages of DR by interacting with endothelial cells (ECs).

In PDR, microglia also contribute to retinal vascular dysfunction. Ben et al. applied scRNA-seq in a diabetic mouse model and found two microglia clusters (37). The cluster more enriched in the DR group was mainly involved in inflammatory responses and exhibited immunoregulatory signatures. Abundant ligand–receptor-paired interactions were found between this microglia cluster and DR-enriched ECs. Notably, the CSF1/CSF1R signaling pathway was discovered between microglia and ECs, leading to inflammation-mediated vascular dysfunction in PDR. CSF1R signaling blockade can attenuate the inflammatory response and vascular dysfunction, which represents a promising therapeutic approach for DR treatment.

### 2.2 Microglia playing double-edged roles in pathological neovascularization

A novel topologically inspired machine learning pipeline based on scRNA-seq data has been developed, finding microglia with inflammasome activation signatures in AMD (38). Kuchroo et al. profiled lesions from postmortem human retinas with AMD and control retinas. During the early stage of AMD, a dry AMD-specific activated microglia cluster appeared with upregulation of APOE, TYROBP, and SPP1, while cell type-specific transcriptional changes exclusively in the subcluster of microglia in late-stage neovascular AMD displayed an inflammasome-related signature including IL1B, NOD2, and NFKB1. CellPhoneDB interaction analysis unveiled significant interactions between this neovascular-specific microglia cluster with pro-angiogenic astrocytes through interleukin-1β (IL-1β) mediating pathological neovascularization in AMD. Therefore, inhibiting microglia-derived IL-1β could provide therapeutic benefits in neovascular AMD. Interestingly, not only did microglia play pathological roles in neovascular AMD, but there also existed a cluster of microglia with antiangiogenic features. Luo et al. compared public scRNA-seq data of retinas from mice with oxygen-induced retinopathy (OIR) with neonatal mouse spine after spinal cord injury, finding a similar cluster of microglia with consistent antiangiogenic characteristics (39). Those microglia expressing antiangiogenic factor TSP-1 made up a limited quantity of microglia in the OIR retina and thus failed to play protective roles in retinal angiogenesis.

### 2.3 Microglia or macrophages: which contributing to the formation of FVMs?

DR can cause preventable vision impairment and blindness due to neovascularization and the formation of vitreous fibrovascular membranes (FVMs) leading to retinal hemorrhage and detachment (40). The cell components of FVMs were considered complex, including retinal glial cells, macrophages/monocytes, fibroblasts, and vascular ECs (41). Hu et al. performed scRNA-seq on surgically harvested PDR FVMs and generated a comprehensive cell atlas of FVM, among which the major cell population was found to be microglia/macrophages (42). Those myeloid cells with marker GPNMB were further identified as activated microglia, differentiated from retina-resident P2RY12^+^ microglia, and increased in PDR setting through pseudotime analysis, presenting both profibrotic and fibrogenic properties. Corano Scheri et al. queried the conclusion in this work but re-analyzed the online deposited raw data and considered monocyte-derived macrophages constituting the majority of immune cells (43). These contradictory results arise from the fact that monocyte-derived macrophages and microglia are indistinguishable by transcriptomic profiling, as microglia may rapidly respond to local insults by downregulating homeostatic microglial markers like P2RY12 and exhibited disease-associated markers such as the expression of macrophage marker APOE (44). Undoubtedly, monocyte-derived macrophages consisted of FVMs in this work, and scRNA-seq data of FVMs from Corano Scheri et al. also found a cluster of macrophages with marker SPP1 expressing proangiogenic genes and potentially contributing to the formation of FVMs (45). The discrepancy between the scRNA-seq data of FVMs in these two studies may be attributed to complex causes. For one thing, the heterogeneity of patients may affect the cell components of FVMs. Patients with different disease histories, for example, whether there existed vitreous hemorrhage or other systematic diseases, can exhibit varied immune cell diversity and responses. For another, different courses of DR may also contribute to this inconsistency. There is consensus that monocyte-derived macrophages activate in the early phase after injury while resident microglia adapt to pathological status in the long term (46). Therefore, further lineage tracing experiments are indispensable to determine the ontogeny of whether microglia or monocyte-derived macrophages dominate in the pathogenesis of FVM.

## 3 Monocyte

Apart from microglia, peripheral immune cells, such as monocytes, can also invade and respond to injuries in the retina. Recent evidence shows that the persistence of monocyte-derived macrophages in both the retina and subretinal space exacerbates pathogenic inflammation, participating in retina degeneration (47).

### 3.1 Monocyte-derived macrophages remaining in retina long term after retinal degeneration

In a mouse model of inducible photoreceptor degeneration, scRNA-seq revealed distinct populations of monocyte-derived macrophages after degeneration (48). Monocyte-derived cells adopted a microglia-like genomic phenotype and remained in the retina. Analysis of scRNA-seq data found a hybrid-like cluster of monocyte-derived macrophages with both pathological microglial and traditional monocytic markers 1 week and 20 days after retinal degeneration, which supported the hypothesis that monocyte-derived macrophages adopted a microglia-like phenotype and remained long-term in the retina suffering altered immune state.

### 3.2 Monocyte-derived macrophages participating in repairing period after optic nerve injury

Optic nerve crush (ONC) injury is characterized by an inflammatory cascade surrounding the optic nerve together with retinal ganglion cells. Two individual studies recently have both uncovered that peripheral monocyte-derived macrophages took part in the inflammatory resolution period after ONC as a supplement to activated resident microglia and border-associated macrophages (BAMs). Benhar et al. generated a scRNA-seq atlas at multiple time points after ONC injury, finding that retinal microglia and astrocytes activated in the early phase and recruited Ccr2^+^ monocytes from the circulation (11). Interactions between chemokines and their receptors were enriched between BAMs, neutrophils, and monocytes, including Ccl2, Ccl3, Ccl4, Ccl6, Ccl12, Cxcr2, and Ccr2. Those Ccr2^+^ monocytes gave rise to Ms4a7^+^ and Gpnmb^+^ macrophage subsets thereafter, characterized by active lipid metabolism and phagocytosis, which were mainly recruited by retinal astrocytes and microglia through Ccl2–Ccr2 signaling axis. Similarly, in the scRNA-seq data from Andries et al., microglia from the injured retina exhibited an elevated expression of the monocyte chemoattractant Ccl2, Ccl3, Ccl4, and Ccl12, recruiting monocyte-derived macrophages to ONC injury and presenting the expression of pro-regenerative secreted factors (49). Ligand–receptor interactions analysis using NicheNet identified the ligands Spp1, Thbs1, Vegfa, and Igf1 in one subset of monocyte-derived macrophages featured with pro-regenerative gene signatures, which are secreted proteins known to promote nerve survival. Therefore, monocyte-derived macrophages may function as a potential responder after ONC to promote axon regrowth and neuron regeneration via paracrine signaling.

### 3.3 Monocytes highly expressing inflammatory markers predicting autoimmune uveitis

Unlike other resident cell types in the retina, monocytes originate from the periphery, which can be isolated and enriched directly from peripheral blood mononuclear cells (PBMCs). ScRNA-seq was applied to encode the landscape of human monocytes from peripheral blood samples of patients with developing VKH disease (50). A disease-specific subset was identified as proinflammatory monocytes. Among differentially expressed genes (DEGs), ISG15 expression in monocytes from VKH patients was upregulated predominantly compared to normal controls. Markedly decreased ISG15 after 3 months of treatment was further functionally validated, suggesting that ISG15 level in circulating monocytes may reflect the treatment response in VKH disease. Similarly, scRNA-seq analyses of monocytes in PBMCs from Behçet’s disease revealed the accumulation of C1Q-high monocytes, the level of which can also aid in monitoring treatment efficacy (51).

## 4 Neutrophil

As first-line defenders against infection and injury, neutrophils can be rapidly recruited to the site of immune response (52). Evidence shows that neutrophils homing into the retina participate in the early stage of retinal diseases such as retinal detachment and AMD (10, 53).

### 4.1 Neutrophils acting as sentinel during rapid injury

Once an injury occurs, neutrophils are “first-line defense” cells arriving at the site of the retina. In an acute retinal arterial ischemia model of *Macaca fascicularis*, Li et al. used scRNA-seq and made bioinformatics analyses just 6 hours after ligation of the ophthalmic artery, in which neutrophils were a non-negligible part in immune cells after rapid ischemic injury (54). Likewise, neutrophils were found infiltrated to the injured retina very soon at the first stage after ONC injury (11, 49). The substantial increase in the number of neutrophils was transient, however; subsequently, macrophages dominated. Further analyses discovered that neutrophil infiltration mainly attributed to the upregulation of chemoattractants in retinal microglia, BAMs, and astrocytes after injury.

### 4.2 Imbalance of unconventional neutrophil subsets determining sex-specific outcome of Behçet’s uveitis

There exist male-biased incidence, severity, and poor prognosis of auto-inflammatory Behçet’s uveitis. Wang et al. recently applied scRNA-seq to circulating neutrophils from healthy individuals and Behçet’s uveitis to elucidate sex-specific heterogeneity in neutrophil composition under normal and auto-inflammatory conditions (55). In healthy individuals, male donors tend to have more inflammatory phenotype neutrophils, while neutrophil-mediated T-cell regulatory functions were relatively active in women. Loss of T-cell regulatory neutrophils was only observed in men under disease conditions, and dysregulation of two unconventional neutrophil (interferon-α responsive and T-cell regulatory) subsets in male patients led to male-biased severity. Interferon-α2a could directly promote unconventional neutrophil subsets to a more immune-regulatory phenotype via Annexin A1 signaling in women, specifically to maintain homeostasis. These findings prompted a promising female-specific sensitive therapy strategy to treat Behçet’s uveitis.

### 4.3 Neutrophil extracellular traps leading to vascular remodeling in retinal neovascularization

An unconventional role of neutrophils in vascular remodeling during late-stage sterile inflammation in OIR mice was found with the utility of scRNA-seq, where neutrophil extracellular traps (NETs) can remodel and clear pathological retinal vasculature through apoptotic elimination of senescent ECs (56). One cluster was identified as neutrophil specially enriched at P17 of OIR when vascular regression was initiated. Gene set variation analysis (GSVA) on scRNA-seq data suggested that neuronal and vascular units have distinct patterns of cellular senescence and secretory phenotypes in OIR, and further experiments confirmed that senescent vascular endothelium triggered the release of NETs. More recently, Dong et al. performed scRNA-seq on the retina–choroid complex from choroidal neovascularization (CNV) mice and found an enhanced Ccl4–Ccr5 interaction from neutrophils to natural killer (NK) cells in the CNV group (57). Their study demonstrated that NK cells interact with neutrophils, converting neutrophils from a pro-senescence state to an anti-senescence state by initiating NET formation, to eradicate senescent cells.

## 5 Discussion

**Figure 1:**
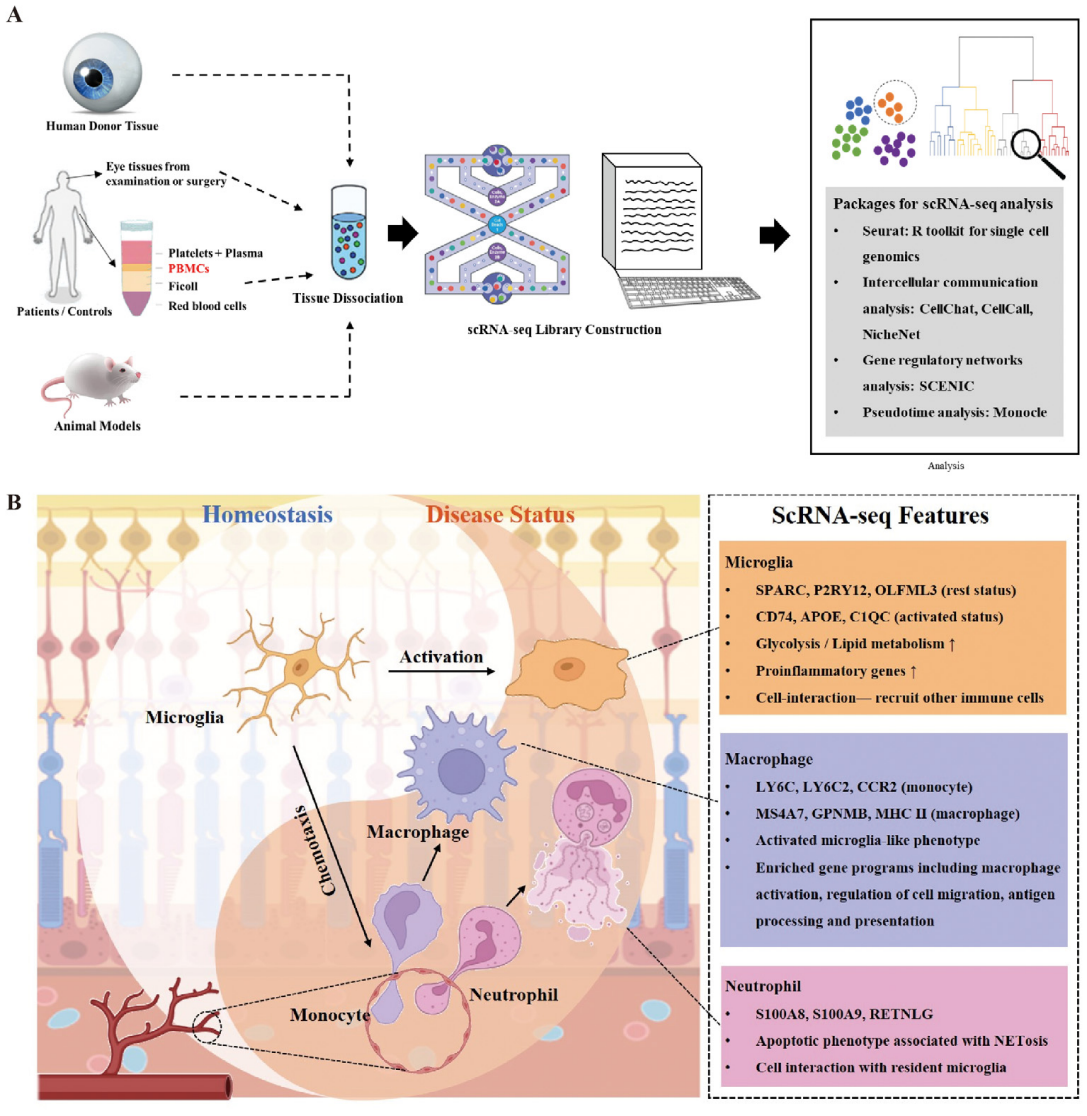
Overview of innate immune cell functions in retina in context of injuries and diseases. **(A)** There are generally four sources of ocular tissue that are used in most single-cell RNA sequencing (scRNA-seq) experiments in the field of ophthalmology, including human postmortem donors, ocular tissues obtained from examinations or surgeries (e.g., aqueous humor), peripheral blood, and animal models. After tissue dissociation, scRNA-seq library is built for further analysis, such as identification of cell clusters and investigation of gene expression using the Seurat package. Packages focusing on intercellular communication, gene regulatory networks, and pseudotime analysis are summarized. **(B)** Main cell types of innate immunity respond to retinal injuries and diseases including resident microglia, monocyte-derived macrophages, and neutrophils infiltrated from circulation. Rest microglia play important roles in defense of retinal homeostasis, which can turn to activated status and recruit other immune cells such as monocytes and neutrophils rapidly at the first stage of injury. Neutrophils as primary defense cells participate in cell interactions with both microglia and monocytes to amplify inflammation. Neutrophil extracellular traps (NETs) from neutrophils have multiple influences on retinal microenvironment. Monocyte-derived macrophages share similar transcriptomes with activated microglia and remain in retina after injury.

Traditional methods and studies usually focus on single-cell type and molecular mechanisms in the context of diseases. Results from RNA-seq are robust. For example, DEGs from microglia by RNA-seq in different disease contexts contained both proinflammatory genes and negative regulator genes, of which a proinflammatory signature was predominant (58). The function of microglia was consequently regarded as promoting inflammation. However, scRNA-seq makes it possible to subdivide microglia into proinflammatory and anti-inflammatory subsets and dissect the protective roles of microglia (36). Therefore, the advent and maturation of the scRNA-seq technique have leveraged the utility of high-throughput sequencing to the single-cell level, thus making it possible to explore pathogenesis on the cellular and molecular levels in the field of ophthalmology (**Figure 1A**).

The ocular innate immune system consists of epithelial barriers, tissue-resident myeloid, and glial cells, which can initiate an inflammatory immune response and result in further recruitment of neutrophils, monocytes, and NK cells in response to infection or injury. Impaired innate immunity participates in the pathobiology of eye diseases (59). Though we mainly focus on microglia, monocytes, and neutrophils in this review, other cell types of the innate immune system are non-negligible. Recent studies applying scRNA-seq found NK cells play important roles in NET formation to inhibit pathological retinal neovascularization, while dendritic cells featured the most active intercellular interactions in HLA-B27-positive uveitis (57, 60). Comprehensive cell atlas in retinal diseases established by scRNA-seq has distinguished classical unremarkable innate immune cells as novel pathogenic cell types and uncovered new pathological mechanisms in innate immune cells throughout the stages of diseases in the retina (**Figure 1B**).

The role of the scRNA-seq technique can be generally divided into three aspects. 1) It acts in a monodrama. ScRNA-seq is an unbiased method to establish a comprehensive cell atlas. 2) It functions as a whistleblower to start the show, which means scRNA-seq can make disease-specific cell types, and DEGs stand out so as to explore new directions for pathogenesis. 3) It has a flexible role throughout the show, meaning it can create a comprehensive cell atlas, analyze DEGs and susceptible pathways, validate cell proportions and gene expression after intervention, explore interactions between cells, and so forth, once needed.

However, there do exist limitations. First, it is known that transcript expressions do not always correlate with protein levels, so it is necessary to validate levels of specific transcripts identified by scRNA-seq at the protein level. Second, analyses of scRNA-seq data may obtain biased and misleading results, as the expensive costs of this technique limit the number of samples in one single study. Substantial samples are needed in statistical analysis to reach a conclusion. Therefore, it is no wonder that different studies applying the scRNA-seq technique in the same pathological condition sometimes found contradictory results (43). A larger number of samples or integration with accessible public scRNA-seq data may reduce the possibility of drawing incorrect conclusions. Third, though scRNA-seq data can reveal the metabolic phenotype of disease-associated cell clusters at the transcriptional level, it is hard to elucidate the cause-and-effect relationship between metabolic phenotype and pathological phenotype. It is confusing whether the metabolic switch of cells causes diseases or whether metabolic changes are adaptations to disease conditions. The efficacy of interfering cells’ metabolic conditions to prevent disease progression remains hazy. ScRNA-seq data can provide clues, while solid evidence requires further experiments to confirm them. A recent study applying scRNA-seq identified activated retinal microglia with metabolic and inflammatory disturbances as the main source of IL-1β in STZ-induced DR mouse retina. Metabolomics analyses further confirmed that activated microglia strongly increased the level of purine metabolism and glycolysis, together with significant metabolism changes in triacylglycerol accumulation. Treatment with both glycolysis and triacylglycerol synthesis inhibitor attenuated IL-1β expression (61). With the rapid development of single-cell multi-omics technologies, integrating transcriptome, genome, epigenome, epitranscriptome, proteome, metabolome, and other omics and acquiring more precise information at the same time have become possible, which may solve these problems (62). A comprehensive single-cell multi-omics atlas of the human retina has been established with a combination of angle-nuclei RNA-seq and single-nuclei ATAC-seq (63). Single-cell multi-omics analyses have uncovered pathogenic genes in eye diseases (64, 65). Further studies should make efforts to integrate transcriptome with proteome or metabolome at the single-cell level to elucidate pathological cause-and-effect relationships behind eye diseases from genotype to phenotype. In addition, it is worth noting that the result of clusterization and annotation of cells may be subjective to some degree with different researchers. For example, it is difficult to distinguish microglia from macrophages, as they share many markers at the transcriptomic level (31, 43, 44). Therefore, it remains technically challenging due to the phenotypic overlap between homologous cells, and validation needs further experiments. More difficultly, identifying the small subset of cellular populations that drive diseases in scRNA-seq data remains a challenge. New methods like Cellular Analysis with Topology and Condensation Homology (CATCH) have been developed to solve this problem (38).

## 6 Conclusion

In the last few years, advancements in scRNA-seq have built a comprehensive cell atlas for multiple eye diseases. Roles of innate immune cells and their complex interactions in eye diseases have been refreshed thanks to the development of scRNA-seq. Emphasis on innate immune cells in eye diseases will be greater with a much deeper elucidation of accessible public scRNA-seq data.
